# Regulatory dynamics of monosomes and polysomes in cellular adaptation

**DOI:** 10.1093/jb/mvag026

**Published:** 2026-04-01

**Authors:** Hirotatsu Imai, Akio Yamashita

**Affiliations:** Department of Biochemistry, Graduate School of Medicine, University of the Ryukyus, 1076 Kiyuna, Ginowan, Okinawa 901-2720, Japan; Division of Molecular Cell Biology, Faculty of Pharmacy, Kindai University, 3-4-1 Kowakae, Higashiosaka, Osaka 577-8502, Japan

**Keywords:** monosome, mRNA, translation, polysome, ribosome

## Abstract

Cytosolic 80S ribosomes have long been considered a uniform translation apparatus, with the only distinction being that they exist as either monosomes or polysomes. However, emerging evidence has revealed that monosomes and polysomes exhibit distinct translational profiles, contributing to selective protein synthesis and localized messenger RNA (mRNA) translation in specific subcellular compartments in mammals. When mammalian cells encounter environmental or metabolic stress, global gene expression is dynamically reprogrammed, accompanied by a marked shift in the monosome-to-polysome ratio, suggesting context-dependent roles for monosome- and polysome-mediated mRNA translation in cellular adaptation. In parallel, accumulating evidence indicates that distinct types of eukaryotic non-translating monosomes are formed under various biological conditions. Moreover, monosomes participate in the first round of mRNA translation on newly synthesized mRNAs, a process coupled with several early events such as nonsense-mediated mRNA decay and messenger ribonucleoprotein remodelling. In this review, we summarize current advances in understanding the roles of monosomes as crucial regulatory layers that shape the proteome across diverse physiological conditions in mammals.

## Graphical Abstract

**Graphical Abstract:**
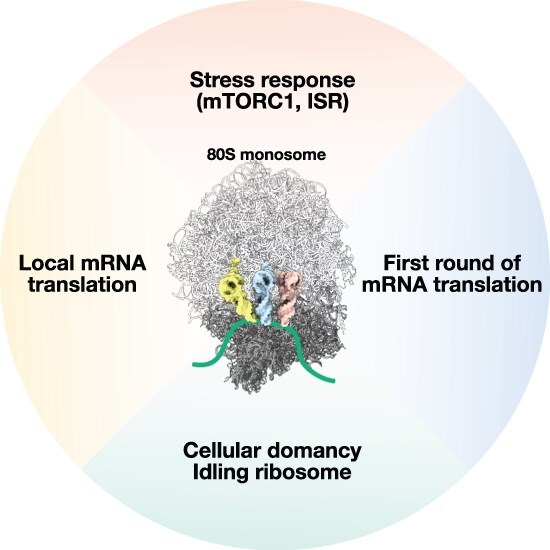

Ribosomes are the fundamental macromolecular machines responsible for translating genetic information into proteins. In eukaryotes, the 60S and 40S ribosomal subunits associate to form the 80S ribosome, which mediates messenger RNA (mRNA) translation in the cytoplasm. Cytosolic 80S ribosomes can be categorized into two major populations: (i) monosomes, which include both single 80S ribosomes translating an mRNA and vacant 80S ribosomes lacking mRNA and (ii) polysomes, in which multiple 80S ribosomes are translating a single mRNA.

Early biochemical analyses using radiolabelled amino acids indicated that protein synthesis occurs predominantly on polysomes. Consequently, monosomes were long regarded merely as post-initiation 80S ribosomes positioned near the start codon, or non-translating vacant 80S ribosomes, rather than actively translating complexes *(**1–3**)*. However, recent translatome analyses have revealed that a large fraction of 80S monosomes are actively translating and significantly contributing to protein synthesis *(**4**)*. In budding yeast, these active monosomes preferentially translate specific mRNAs, such as those containing short open reading frames (ORFs) or upstream ORFs (uORFs), as well as low-abundance but functionally important mRNAs encoding transcription factors, kinases and phosphatases *(**4**)*.

The physiological relevance of monosome-mediated mRNA translation is particularly evident in the mammalian nervous system. Recent studies have demonstrated that monosome-mediated mRNA translation occurs in specific subcellular compartments, such as neuronal axons and dendritic spines *(**5**,*  *6**)*. Notably, while highly abundant synaptic proteins like Camk2a and PSD95 are predominantly translated by polysomes, a diverse set of mRNAs encoding other key proteins involved in synaptic function and plasticity is preferentially translated by monosomes in these local domains. These findings suggest that monosome translation serves as a crucial regulatory mechanism that confers both target selectivity and spatial specificity to the proteome.

Many lines of evidence have shown that the relative proportion of 80S monosomes and polysomes varies substantially across cell types, differentiation stages and in response to intracellular and extracellular stimuli (Fig. 1) *(**7–9**)*. For instance, various environmental and metabolic stressors that inhibit the mechanistic target of rapamycin complex 1 (mTORC1) signalling or induce the integrated stress response (ISR) result in a marked polysome collapse and a concomitant increase in the 80S monosome population *(**10–14**)*. In addition, during mTORC1 inhibition, the accumulating monosomes comprise not only actively translating monosomes but also non-translating 80S monosome complexes. This dynamic reprogramming of the translational machinery raises a fundamental question: what is the physiological significance of such a shift in the monosome-to-polysome ratio under these specific conditions? In this context, the 80S ribosome should no longer be viewed as a uniform translation apparatus, but rather as a dynamically allocated platform that exists in multiple functional states.

**Fig. 1:**
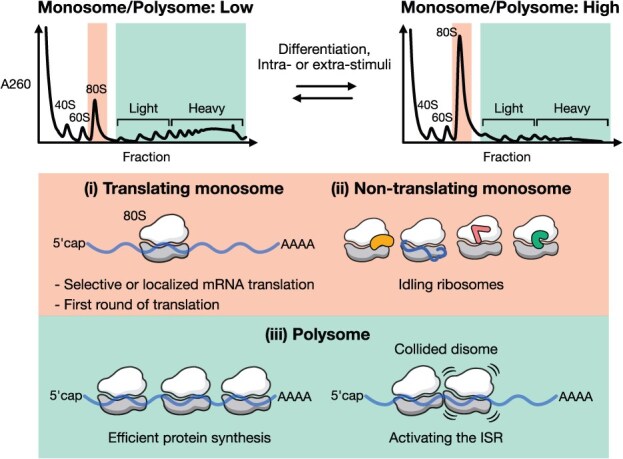
**Monosomes and polysomes in mammals.** Cytosolic ribosomes are categorized into monosomes and polysomes. The monosome-to-polysome ratio varies according to cellular differentiation status and environmental conditions. Under conditions that shift the monosome-to-polysome ratio from low to high, a decrease in polysome abundance is particularly evident in the heavy polysome fractions. Monosomes are further classified into (i) actively translating monosomes and (ii) non-translating monosomes. Actively translating monosomes engage in the selective translation of specific mRNAs. In the nervous system, monosomes are involved in selective and localized mRNA translation. Actively translating monosomes also represent the first round of translation, where newly synthesized mRNAs are initially translated by a single ribosome before the formation of polysomes for efficient protein synthesis (iii, left). Through this process, translation-dependent mRNA quality control mechanisms, such as NMD and RMD, and the remodelling of mRNPs occur. During polysome-mediated translation, when ribosomes stall at specific codons, trailing ribosomes collide with the leading ribosomes, resulting in the formation of collided disomes (iii, right). These ribosome collisions induce the ISR. On the other hand, non-translating monosomes (ii) include vacant 80S ribosomes lacking mRNA and those associated with various types of monosome-binding proteins. The relative proportions of these ribosomal populations are dynamically altered in response to physiological and stress conditions.

## mTORC1 Signalling

Cell growth is an energetically demanding process and is tightly regulated in response to the availability of nutrients and growth stimuli. In mammals, mTORC1 serves as a central regulator of this process. mTORC1 promotes cap-dependent translation initiation through the direct phosphorylation of eukaryotic initiation factor 4E-binding protein (4EBP) and ribosomal protein S6 kinase (S6K) *(**15**)*. Under nutrient deprivation or upon rapamycin treatment, the inhibition of mTORC1 leads to decreased phosphorylation of 4EBP and S6K, resulting in the repression of translation initiation and a marked shift in ribosome distribution from polysomes to monosomes *(**14**,*  *16**)*.

mTORC1 plays an important role in the translation of 5′-terminal oligopyrimidine (TOP) mRNAs, which encode ribosomal proteins and translation factors *(**17**,*  *18**)*. This regulation is mediated by the RNA-binding protein La-related protein 1 (LARP1), a direct phosphorylation substrate of mTORC1. Upon mTORC1 inactivation, dephosphorylated LARP1 binds to the TOP motif of target mRNAs with high affinity, competing with eukaryotic initiation factor 4E (eIF4E) for cap binding and thereby blocking translation initiation *(**19–23**)*. Recent findings suggest the possibility that LARP1-mediated regulation of TOP mRNAs involves the formation of LARP1–TOP mRNA–ribosome complexes. Under normal growth conditions, a subset of TOP mRNAs associates with LARP1 and 40S subunits to form TOP–40S complexes *(**24**)*. When mTORC1 is inhibited, the 60S subunit binds to the TOP–40S complex to form the TOP–80S complex, which then shifts to the monosome fraction *(**25–28**)*. Although LARP1 plays a central role in the translational repression and stabilization of TOP mRNAs within these TOP–40S/80S complexes, the functional role of the associated 40S and 80S ribosomes remains under debate. One study proposed that the TOP–80S complex exhibits monosome translation at low levels, potentially contributing to TOP mRNA stability during mTORC1 inhibition *(**27**)*. In contrast, another study identified a ribosome-binding region (RBR) within LARP1 that directly associates with the 40S subunit and occludes the mRNA entry channel *(**28**)*. The LARP1–RBR mutant impaired the shift of TOP mRNAs to the monosome fraction, suggesting that ribosomes within TOP–80S complexes may directly bind to LARP1 rather than to the mRNA. Intriguingly, however, the RBR mutant retained the ability to repress translation and stabilize TOP mRNAs upon mTORC1 inhibition, indicating that these principal functions of LARP1 do not require direct ribosome binding. Thus, the functional significance of ribosome association within TOP–40S/80S complexes remains unclear.

## Integrated Stress Response

The ISR is a translational reprogramming pathway activated by diverse cellular stresses, including endoplasmic reticulum (ER) stress, amino acid deprivation, viral infection and oxidative stress. These stress signals activate eIF2α kinases (PERK, GCN2, PKR and HRI), which phosphorylate eIF2α at Ser51 and thereby inhibit the guanine nucleotide exchange factor (GEF) activity of eIF2B *(**29**,*  *30**)*. As a result, the availability of the eIF2–GTP–Met–tRNA_i_ ternary complex is limited, leading to reduced formation of the 43S pre-initiation complex and a global repression of cap-dependent translation initiation. Similar to mTORC1 inhibition, the ISR causes a marked shift in ribosome distribution from polysomes to monosomes *(**10–13**)*.

Despite the global suppression of mRNA translation during the ISR, cells must selectively maintain the synthesis of stress adaptation proteins, such as activating transcription factor 4 (ATF4) and C/EBP homologous protein (CHOP). mRNAs encoding ATF4 and CHOP contain uORFs in their 5′-untranslated regions (UTRs) that promote translation of the main ORF when the availability of the eIF2–GTP–Met–tRNA_i_ ternary complex is limited. These mRNAs undergo a stress-induced shift from monosomes and light polysomes to heavy polysomes, resulting in increased protein production *(**10**,*  *12**,*  *31**)*.

In the ISR, large fractions of mRNAs dissociate from polysomes, leading to the accumulation of non-translating mRNAs in the cytoplasm. Multivalent interactions among these mRNAs and RNA-binding proteins, such as GAP SH3-binding protein (G3BP) drive liquid–liquid phase separation and the formation of stress granules (SGs). SGs are enriched in 40S subunits and several translation initiation factors, including eIF4E, eIF4G and eIF3, while largely excluding 60S subunits and elongating 80S ribosomes *(**32**)*. Importantly, mRNA recruitment to SGs is selective. While long mRNAs and those encoding housekeeping proteins are efficiently incorporated into the SGs, mRNAs encoding molecular chaperones and stress-adaptive ATF4 and CHOP are typically excluded *(**33**)*. Recent studies have shown that a key determinant of this selectivity is the presence of 80S ribosomes bound to an mRNA. mRNAs associated with one or more 80S ribosomes are prevented from entering SGs *(**34–37**)*. uORFs in ATF4 and CHOP mRNAs function as binding sites for 80S ribosomes, in addition to their canonical role in promoting main ORF translation, thereby excluding these transcripts from SGs *(**36**)*. Thus, monosomes formed on uORFs during the ISR act as platforms that regulate mRNA localization between the cytoplasm and SGs and facilitate translation of the main ORF.

Recently, it has been increasingly recognized that ribosome collisions resulting from translational stalling serve as a trigger for the activation of the ISR. While acute amino acid starvation activates the eIF2α kinase GCN2 primarily via the direct binding of accumulating uncharged tRNAs *(**38**,*  *39**)*, stress conditions that do not elicit appreciable accumulation of uncharged tRNAs, such as depletion of specific tRNAs or treatment with translation elongation inhibitors, cause elongating ribosomes to stall at specific codons *(**39**,*  *40**)*. Subsequently, the trailing ribosome collides with the leading stalled ribosome, resulting in the formation of collided ribosomes, termed disomes *(**41**,*  *42**)*. These disomes act as scaffolds for the activation of GCN2. Structural studies have revealed that the GCN2 coactivator GCN1 specifically recognizes a disome by interacting with both the leading and colliding ribosomes, including direct interactions with the ribosomal P-stalk base, and subsequently recruits GCN2 *(**43**)*. The ribosomal P-stalk canonically recruits translational GTPases (trGTPases) via its conserved C-terminal regions and promotes translation elongation *(**44–46**)*. Upon GCN2 recruitment to the disome, direct engagement with these C-terminal regions stimulates GCN2 kinase activity *(**47**)*. In light of this, it has been proposed that while the P-stalk is occupied by cycling trGTPases under normal translation, unknown mechanisms upon ribosomal stalling might liberate the P-stalk, enabling GCN2 to access the exposed C-terminal regions. Alternatively, or additionally, ribosome collisions engage the mitogen-activated protein kinase kinase kinase (MAPKKK) ZAKα to facilitate GCN2 activation. Notably, an ATP-binding defective mutant of ZAKα was still able to support eIF2α phosphorylation in response to ribosome collisions, suggesting that the role of ZAKα in this case may be structural or binding-related rather than catalytic *(**48**)*. The activation of GCN2 on collided ribosomes leads to eIF2α phosphorylation and the ISR. Since ribosome collisions inherently require the presence of at least two ribosomes on a single mRNA, this ribosome collision-dependent ISR induction is thought to occur primarily on polysomes rather than monosomes.

## Selective and Local mRNA Translation

In highly polarized cells, such as neurons, local mRNA translation within specialized subcellular compartments like dendrites and axons is essential for maintaining complex morphology and synaptic plasticity. Due to the spatial constraints within tiny synaptic compartments, such as dendritic spines (<0.1 μm^3^), the accommodation of large polysome complexes (~100 to 200 nm) is physically limited *(**49**,*  *50**)*. Consequently, a large fraction of actively translating 80S monosomes acts as a major source of local protein synthesis *(**5**,*  *6**)*. Polysome profiling and RNA-seq have demonstrated that many localized mRNAs, particularly those encoding key synaptic proteins, are preferentially translated by monosomes in the synaptic neuropil *(**5**)*.

The selectivity for an mRNA to be translated by monosomes or polysomes is largely dictated by the inherent features of the transcript itself. In the nervous system, transcripts that are preferentially translated by monosomes exhibit specific features, including longer ORFs, longer 5′UTRs, the presence of uORFs, lower translation initiation rates and lower codon optimality *(**5**)*. These features generally attenuate translation initiation efficiency or confer moderate initiation kinetics, which restricts the consecutive loading of multiple ribosomes and instead favors active elongation by single ribosomes. Of note, several features determining monosome preference in mammals do not align with those determined in budding yeast. In budding yeast, monosomes have been shown to preferentially translate transcripts with short ORFs and mRNAs targeted by nonsense-mediated mRNA decay (NMD) *(**4**)*. The contrasting observation in the mammalian neuropil, where monosome occupancy positively correlates with longer ORFs, might be explained by the structural expansion and increased complexity of mRNAs during evolution from lower to higher eukaryotes *(**5**)*.

In addition, the selectivity for monosome or polysome translation may also arise from compositional differences within the ribosomes themselves. Recent studies have revealed that the protein composition of ribosomes is not necessarily uniform across tissues or even within a clonal cell, an emerging concept known as ribosome heterogeneity. Since such heterogeneity has been shown to alter the translation efficiency of selective subsets of mRNAs *(**51–55**)*, it is possible that the preference of a given transcript for monosomes or polysomes is partly dictated by this heterogeneous ribosome composition.

## Non-Translating Idling Monosome

**Fig. 2:**
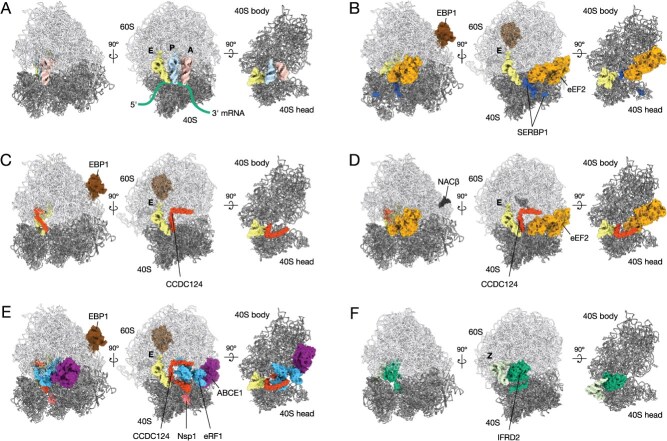
**Mammalian translating or non-translating 80S ribosomes.** (A–F) Structural models of the mammalian translating or non-translating 80S ribosomes determined by cryo-EM or cryo-ET. Each model is shown from the front (centre panel), side (left panel) and top (right panel). (A) *In situ* cryo-ET structure of the human translating 80S ribosome bound to A-site tRNA (light pink), P-site tRNA (light blue) and E-site tRNA (light yellow) (PDB: 9P7Y), along with an illustration of the presumed mRNA (green line). (B) Cryo-EM structure of the human 80S ribosome bound to SERBP1 (blue), eEF2 (orange), EBP1 (brown) and E-site tRNA (light yellow) (PDB: 6Z6M). (C) Cryo-EM structure of the human 80S ribosome bound to CCDC124 (red), EBP1 (brown) and E-site tRNA (light yellow) (PDB: 6Z6L). (D) *In situ* cryo-ET structure of the human 80S ribosome bound to CCDC124 (red), eEF2 (orange), NACβ (black) and E-site tRNA (light yellow) (PDB: 9P9H). (E) Cryo-EM structure of the human 80S ribosome bound to CCDC124 (red), eRF1 (cyan), ABCE1 (purple), EBP1 (black), E-site tRNA (light yellow) and SARS-CoV-2 Nsp1 (pink) (PDB: 6ZME). An atomic model of the C-terminus of CCDC124 is included. (F) Cryo-EM structure of the rabbit 80S ribosome bound to IFRD2 (green) and Z-site tRNA (light green) (PDB: 6MTC).

While the importance of monosome translation has become increasingly appreciated, it is evident that not all monosomes are actively translating. Under specific physiological conditions, such as nutrient deprivation or cellular dormancy, a subset of monosomes associates with several monosome-binding proteins, forming non-translating idling 80S ribosomes (also referred to as dormant, hibernating, inactive, or silent ribosomes) in an mRNA-independent manner *(**56**,*  *57**)*. Although these complexes have been most extensively characterized in budding yeast *(**58–63**)*, recent advances in cryo-electron microscopy (cryo-EM) and *in situ* cryo-electron tomography (cryo-ET) have enabled the classification of diverse non-translating idling ribosomes across various eukaryotes, including mammals, flies, fish and birds (Fig. 2) *(**9**,*  *62–69**)*.

Among the eukaryotic hibernation factors, suppressor of target of Myb 1 (Stm1) in budding yeast is well characterized. Under conditions of glucose or amino acid starvation, Stm1 binds to the mRNA entry channel of the 40S subunit and promotes the formation of idling 80S monosomes *(**70**)*. Stm1-bound 80S monosomes stabilize the existing ribosome pool during nutrient depletion and facilitate the rapid resumption of translation upon nutrient repletion *(**14**,*  *60**)*. Given that ribosome biogenesis requires multiple energy-consuming steps, including rRNA transcription, processing, modification and assembly with ribosomal proteins, temporarily preserving pre-existing ribosomes during starvation represents an energetically efficient strategy for maintaining the translational machinery. Recently, Stm1 has also been reported to interact with the transcription factor Ifh1 to positively regulate ribosome biogenesis, highlighting its dual roles in coordinating ribosome synthesis and degradation according to nutrient availability *(**71**)*.

In mammals, SERPINE1 mRNA-binding protein 1 (SERBP1) promotes non-translating monosome formation. SERBP1 is an ortholog of Stm1 and directly binds to the mRNA entry channel and extends through the A and P sites of the 40S subunit (Fig. 2A and B). Notably, SERBP1 often binds to the 80S ribosome together with eukaryotic elongation factor 2 (eEF2), a typical trGTPase, which promotes the translocation of the mRNA and tRNAs during translation elongation. In SERBP1/eEF2-bound monosomes, eEF2 is observed in its GDP-bound form. Given that eEF2 immediately dissociates from the ribosome after GTP hydrolysis, SERBP1 might anchor eEF2–GDP on the 80S ribosome. Although the upstream signals that trigger SERBP1 binding remain incompletely understood, mTORC1 and eukaryotic elongation factor 2 kinase (eEF2K) have been implicated in this process *(**14**,*  *66**)*. mTORC1 can directly phosphorylate SERBP1 *in vitro* and also acts upstream of eEF2K, which suggests that mTORC1 inactivation triggers the assembly of SERBP1/eEF2-bound monosomes. Moreover, SERBP1/eEF2-bound 80S monosomes have been detected in rabbit reticulocyte lysates (RRLs), indicating that the formation of idling ribosomes may be linked to the overall translational state of the cell *(**65**)*.

In addition to SERBP1, coiled-coil domain-containing protein 124 (CCDC124) (Lso2 in budding yeast) has also been identified as a ribosome hibernation factor in mammals. CCDC124 was found to associate with 80S ribosomes purified from confluent HEK293T cells, where it bridges the decoding centre of the 40S subunit and the GTPase-associated centre (GAC) of the 60S subunit, occupying the A and P sites (Fig. 2C) *(**63**)*. Recent *in situ* cryo-ET of stressed HEK293 cells revealed an in-cell structure in which CCDC124 and eEF2 are simultaneously bound to 80S ribosomes (Fig. 2D) *(**72**,*  *73**)*. Furthermore, CCDC124 associates with eukaryotic peptide chain release factor 1 (eRF1), ATP-binding cassette sub-family E member 1 (ABCE1) and SARS-CoV-2 Nsp1 (Fig. 2E). In this structure, the C-terminus of CCDC124 binds to the cleft formed between the 40S head and body upon SARS-CoV-2 Nsp1 binding. These observations imply that CCDC124 interacts with multiple functional partners and that its binding mode to the 40S subunit is likely modulated by these auxiliary proteins.

Interferon-related developmental regulator 2 (IFRD2) was also identified as a hibernation factor in RRLs *(**65**)*. IFRD2 binds across the P and E sites of the ribosome and inserts its C-terminal helix into the mRNA channel of the 40S subunit, thereby stabilizing an idling state (Fig. 2F). In IFRD2-bound 80S monosomes from RRLs, a tRNA molecule has been observed at an atypical Z-site, although it appears not to be strictly required for IFRD2 binding to human ribosomes.

In addition to the tRNA binding sites and the mRNA tunnel, certain factors associate near the peptide exit tunnel of the 60S subunit. Several cryo-EM and cryo-ET structures show that ErbB3-binding protein 1 (EBP1) and the β-subunit of nascent polypeptide-associated complex (NACβ) bind near the peptide exit tunnel (Fig. 2B–E) *(**63**,*  *73**,*  *74**)*. However, their specific roles in idling ribosomes are not yet fully understood. By contrast, in idling ribosomes from zebrafish eggs, death-associated protein 1b (Dap1b) occupies the peptide exit tunnel near the peptidyl transferase centre (PTC) together with eIF5A *(**67**)*. Notably, Dap1b can bind to mammalian ribosomes and suppress *in vitro* translation in RRLs *(**67**)*. Although it remains unknown whether the mammalian ortholog, DAPL1, plays a similar role *in vivo*, it may function under specific physiological conditions that necessitate more stable non-translating ribosome complexes in mammals. Collectively, these structural observations suggest that these ribosome-binding proteins participate in modulating ribosome activity under specific physiological or stress conditions, although their precise regulatory roles remain to be fully elucidated.

Across various eukaryotes, including mammals, flies, fish and birds, multiple types of idling ribosomes appear to exist, reflecting a diverse range of translationally inactive states. However, the molecular mechanisms, physiological relevance and functional relationships among these distinct idling ribosome complexes remain incompletely understood. For instance, inhibition of mTORC1 or activation of eEF2K has been reported to promote the formation of SERBP1-bound idling ribosomes. Yet, it is still unclear at which stage of translation active ribosomes transition into an idling state, or whether such complexes are instead reassembled after ribosome recycling. Moreover, the factors responsible for reactivating idling ribosomes and returning them to active translation remain unknown. In yeast, the Dom34–Hbs1 complex has been shown to efficiently dissociate Lso2-bound 80S monosomes, whereas Stm1-bound monosomes appear resistant to this recycling system *(**63**)*. Whether their mammalian orthologs display similar target specificities has not yet been established. The boundary between translational pausing and hibernation also remains ambiguous. Cryo-EM studies have visualized idling ribosomes that retain one or more tRNAs, yet it is often difficult to determine whether such structures represent transient elongation arrest or stably inactivated states. An additional question is whether hibernation factors themselves possess intrinsic inhibitory activity toward ribosomes, independent of translational repression induced by mTORC1 inhibition or eIF2α phosphorylation. For example, similar to yeast Stm1 *(**61**)*, excess Dap1b, which stably inserts into the polypeptide exit tunnel of the zebrafish 80S ribosome, has been reported to inhibit *in vitro* translation *(**67**)*. However, it remains to be determined whether other monosome-binding proteins exert similar inhibitory effects. Further studies will be necessary to clarify whether each hibernation factor actively suppresses translation on its own or instead functions cooperatively with other cellular components to maintain the idling state.

## Translation of Newly Synthesized mRNAs

After export from the nucleus to the cytosol, newly synthesized mRNAs are initially translated by a single ribosome as a translating monosome (referred to as the first round of translation), before additional ribosomes can be loaded to form polysomes. At this stage, two translation-coupled events are triggered: (i) the remodelling of mRNPs for steady-state translation *(**75**)*, and (ii) translation-dependent programmed mRNA degradation, such as mRNA surveillance (*e.g.* NMD) and Regnase-1–mediated mRNA decay (RMD) *(**76**,*  *77**)*. Thus, the monosome state serves as a critical checkpoint that determines whether a newly synthesized mRNA proceeds to polysome-mediated steady-state translation or is instead degraded.

The mRNP remodelling includes the replacement of the cap-binding proteins (from the CBP20–CBP80 complex to the eIF4E–eIF4A–eIF4G complex) and poly(A)-binding proteins (from PABPN to PABPC), the displacement of EJCs and mRNA circularization. As a consequence, pre-existing translating mRNAs and newly synthesized mRNAs may differ in the composition and architecture of their mRNP complexes, which could in turn influence the efficiency of ribosome recruitment, and thus the transition from monosome to polysome, as well as their susceptibility to RNA decay or translational repression. Consistent with this idea, translatome analysis of 4-thiouridine-labeled newly synthesized mRNAs using P-TRAP-seq (P-stalk-mediated translating ribosome affinity purification) in ER-stressed HEK293 cells has shown that newly synthesized mRNAs transcribed following ER stress tend to escape phospho-eIF2α-dependent translational repression compared with pre-existing mRNAs *(**78**)*. Although the molecular mechanism underlying the difference in translational efficiency between newly synthesized and pre-existing mRNAs remains unclear, one possible explanation is a difference in cap-binding proteins (*i.e.* the CBP20–CBP80 complex vs. the eIF4E–eIF4A–eIF4G complex), which may affect the recruitment efficiency of the 43S pre-initiation complex. Of note, the replacement of CBP20 with eIF4E has been demonstrated in budding yeast *(**79**,*  *80**)*, whereas in mammalian cells, this process remains controversial and awaits further investigation *(**81**,*  *82**)*. In addition, differences in poly(A) tail length between newly synthesized and pre-existing mRNAs have also been implicated in translational control. During acute ER stress, newly synthesized *XBP1* mRNAs with long poly(A) tails escape translational repression, whereas pre-existing *XBP1* mRNAs with short poly(A) tails are translationally repressed *(**83**)*. Together, these findings suggest that the maturation state and temporal origin of mRNAs can modulate their translational responsiveness under stress conditions, potentially contributing to the distinct translational behaviours of monosome- and polysome-associated mRNAs.

## Concluding Remarks

Recent advances have revealed that mammalian 80S ribosomes exist across a spectrum of functional states that extend far beyond the classical distinction between monosomes and polysomes. Actively translating monosomes contribute to selective and localized protein synthesis, whereas stress conditions, such as mTORC1 inhibition or ISR activation trigger a large-scale redistribution of ribosomes. This shift includes the increased formation of specific ribosome–mRNA complexes, such as TOP–80S complexes, and structurally distinct, mRNA-free idling monosomes. These findings suggest that ribosome allocation is dynamically regulated in response to cellular context. Given the emerging links between ribosome dormancy and diverse physiological and pathological contexts, including development, viral infection, aging and disease, dissecting the molecular basis of mammalian idling ribosomes represents an important frontier in translation research. Clarifying whether ribosome idling reflects a transient pause within the translational cycle or a distinct branch of ribosome fate will be essential for understanding translational homeostasis. Viewing the 80S ribosome not as a static apparatus but as a dynamic state-switching platform may fundamentally reshape our understanding of translational regulation in mammals.
